# The importance of direct and indirect trophic interactions in determining the presence of a locally rare day-flying moth

**DOI:** 10.1007/s00442-021-05100-9

**Published:** 2022-01-08

**Authors:** Hagen M. O’Neill, Sean D. Twiss, Philip A. Stephens, Tom H. E. Mason, Nils Ryrholm, Joseph Burman

**Affiliations:** 1 Centre for Environmental Research Innovation and Sustainability, Institute of Technology Sligo, Ash Lane, Sligo, Ireland; 2 Department of Biosciences, Durham University, South Road, Durham, DH1 3LE UK; 3 Swiss Ornithological Institute, Seerose 1, CH-6204 Sempach, Switzerland; 4 Department of Electronics, Mathematics and Natural Sciences, Faculty of Engineering and 44 Sustainable Development, University of Gävle, Gävle, Sweden; 5 Ecology Research Group, Canterbury Christ Church University, Canterbury, Kent, England

**Keywords:** Conservation ecology, Ecosystem engineering, Ecosystem engineers, Trophic effects, Invertebrates, Lepidoptera, Red deer, *Zygaena purpuralis*

## Abstract

Ecosystem engineers affect other organisms by creating, maintaining or modifying habitats, potentially supporting species of conservation concern. However, it is important to consider these interactions alongside non-engineering trophic pathways. We investigated the relative importance of trophic and non-trophic effects of an ecosystem engineer, red deer, on a locally rare moth, the transparent burnet (*Zygaena purpuralis*). This species requires specific microhabitat conditions, including the foodplant, thyme, and bare soil for egg-laying. The relative importance of grazing (i.e., trophic effect of modifying microhabitat) and trampling (i.e., non-trophic effect of exposing bare soil) by red deer on transparent burnet abundance is unknown. We tested for these effects using a novel method of placing pheromone-baited funnel traps in the field. Imago abundance throughout the flight season was related to plant composition, diversity and structure at various scales around each trap. Indirect effects of red deer activity were accounted for by testing red deer pellet and trail presence against imago abundance. Imago abundance was positively associated with thyme and plant diversity, whilst negatively associated with velvet grass and heather species cover. The presence of red deer pellets and trails were positively associated with imago abundance. The use of these sites by red deer aids the transparent burnet population via appropriate levels of grazing and the provision of a key habitat condition, bare soil, in the form of deer trails. This study shows that understanding how both trophic and non-trophic interactions affect the abundance of a species provides valuable insights regarding conservation objectives.

**Supplementary Information:**

The online version contains supplementary material available at 10.1007/s00442-021-05100-9.

## Introduction

A critical goal of conservation ecology is to investigate the mechanisms that contribute to the abundance and distribution of a species in decline to prevent extirpation (Gunn and Caughley 1995). To achieve this, major ecological determinants of species persistence are investigated, such as resource availability, habitat suitability, con- and hetero-specific competition and predation (Chapman and Reiss 1998). As these ecological processes become better understood, conservation ecologists can make informed decisions on prioritizing conservation efforts.

One potential determinant of a species’ dynamics that has been increasingly acknowledged in conservation ecology results from the actions of ecosystem engineers (Barbosa et al. 2019; Romero et al. 2015). Ecosystem engineers are organisms that alter the biotic or abiotic components of an ecosystem through direct or indirect means, thereby creating, modifying, or maintaining habitat condition and resource availability for other species (Jones et al. 1994). Well-documented examples of ecosystem engineers include African bush elephants (*Loxodonta africana*), beavers (*Castor spp*.), and termites (Infraorder Isoptera), all of which significantly alter their ecosystems (Barry et al. 2019; Melis et al. 2006).

One of the principal effects of ecosystem engineers arises through their modification or creation of hetero-specific habitat (Bangert and Slobodchikoff 2006). For example, wild boar (*Sus scrofa*) rooting behaviour reduces gramminoid encroachment and creates suitable larval microhabitat for the grizzled skipper (*Pyrgus malvae*) in heathland and grasslands (De Schaetzen et al. 2018). Similarly, anthills created by yellow meadow ants (*Lasius flavus*) indirectly provide suitable microhabitat conditions and promote hostplant growth for the larval development of transparent burnets (*Zygaena purpuralis*) in calcareous grasslands (Streitberger and Fartmann 2015). If a species relies on the physical modifications enabled by an ecosystem engineer and would otherwise decline, then the ecosystem engineer and its associated effects are clearly of conservation interest (Crain and Bertness 2006).

Burnet moths (*Zygaena* Fabricius 1775) suffer from continued habitat loss throughout Britain. (Sarin and Bergman 2010), yet the ecology and behaviour of burnets is poorly understood in comparison to butterflies (Bourn 1995; Hofmann and Tremewan 2010). Generally, zygaenids inhabit dry grasslands, and semi-natural pastures are an important habitat in particular (Franzén and Ranius 2004). In Britain, exceptionally favourable habitat conditions exist on the Hebridean islands in west Scotland, where the richness of *Zygaena* is attributed to the presence of specific microhabitats (Bourn 1995). Transparent burnets (*Z. purpuralis*) depend upon south-facing basalt outcrops that influence the growth of herb-rich vegetation communities that support wild thyme (*Thymus serpyllum*), their foodplant (Ravenscroft and Young 1996). Whilst it is generally accepted that transparent burnets lay egg batches on low-lying forbs in bare soil and sheltered hollows on the ground (Bourn 1995), the importance of vegetation composition, diversity and structure are unknown. These herb-rich, short-sward grasslands are exclusively grazed by free ranging red deer (*Cervus elaphus*), yet the nature of their role in currently modifying burnet habitat is currently unknown (O'Neill 2017). Ungulate grazing can benefit arthropod communities by suppressing competition to hostplants and by promoting suitable microhabitat conditions and vegetation structures (WallisDeVries et al. 2016). Furthermore, these grasslands are steep coastal slopes therefore resulting in a low topsoil layer, and much of the bare soil found on these slopes is a result of red deer movements, forming deer trails (Clutton-Brock et al. 1982). This suggests that deer could be providing suitable microhabitat requirments for transparent burnet egg-laying.

The aims of this research were to evaluate the effects of direct trophic interactions (effects of red deer grazing on plant composition, diversity and structure) and indirect trophic interactions (ecosystem engineering as a function of red deer activities) on transparent burnet abundance. Ecosystem engineers not only physically modify the environment, but also belong to the food web and accounting for their non-engineering trophic pathways is critical in understanding how ecosystem engineers affect other species (Prugh and Brashares 2012; Sanders et al. 2014; Wootton 2002). For direct trophic effects, we aimed to establish the importance of specific local habitat characteristics such as plant composition, diversity and structure that promote transparent burnet densities. The ecosystem engineering role of red deer by indirectly providing beneficial habitat for the moth was examined by testing for associations between red deer trails and moth abundance. The importance of these conditions were examined quantitatively at a range of ecologically relevant spatial scales, to understand the critical scales at which favourable habitat drives moth abundances.

## Materials and methods

### Study species

Transparent burnets are day-flying, aposematic moths with a Palaearctic distribution (Niehuis et al. 2007; Tremewan 1985). Transparent burnets typically display limited mobility, and occupy small areas (Tremewan 1985). When suitable habitat is present, species can occur abundantly and tend to be dominant pollinators (Franzén and Ranius 2004). Transparent burnets have an univoltine imago form, occuring from early June to July, and inhabit steep, south-facing grassy slopes on coastal cliffs or inland limestone areas (Tremewan 1985) that feature wild thyme, the larval hostplant and favoured adult foodplant (Bourn 1995; Wormell 1983). The species forms metapopulations of several small, interconnected colonies that are susceptible to increased isolation via landscape fragmentation (Franzén and Nilsson 2012). The west-coast of Scotland now features the largest metapopulation of transparent burnets in the British Isles, as the species has been extirpated from most of its former range (Bourn 1995; Tremewan 1985).

### Study region

**Fig. 1:**
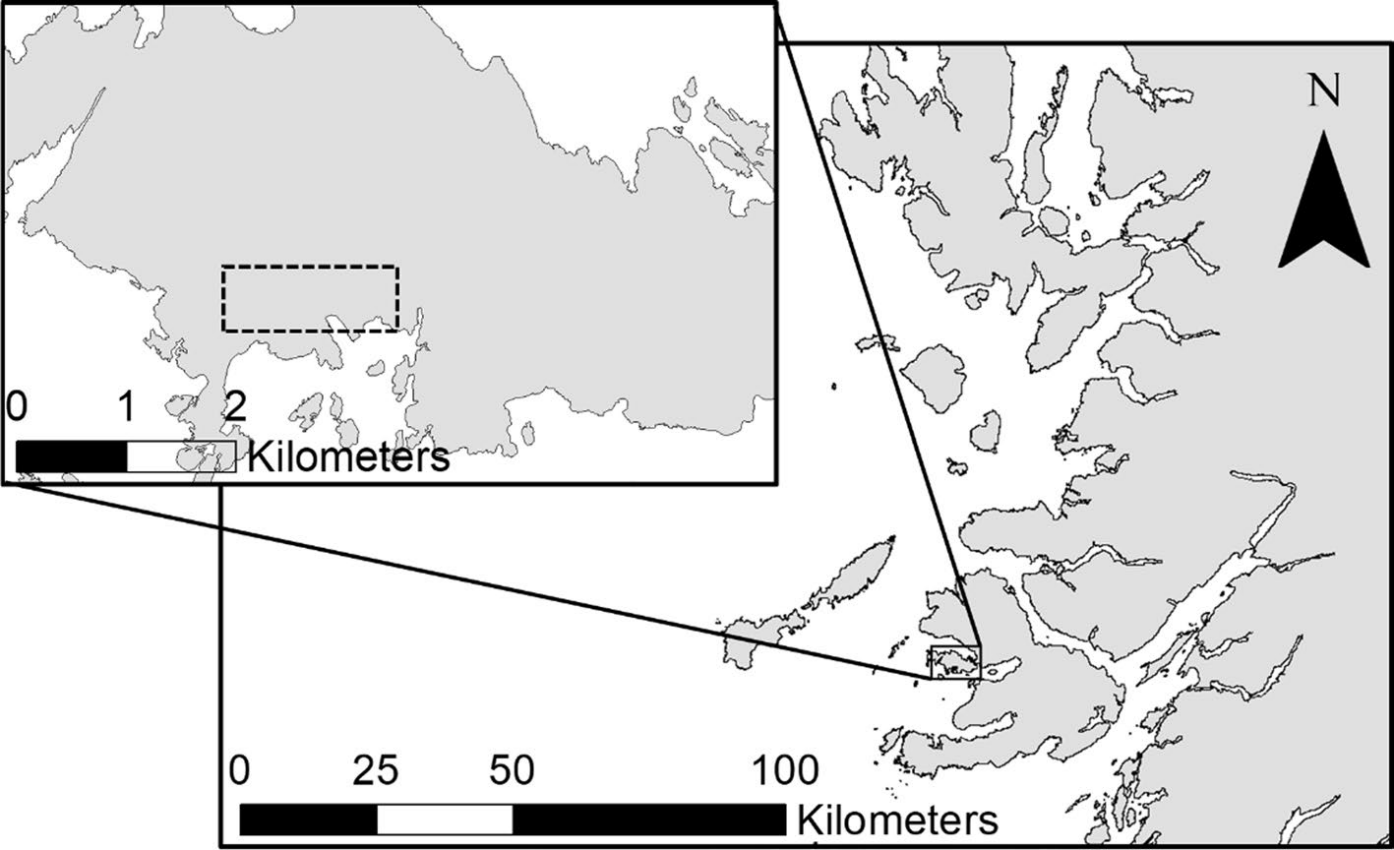
The Isle of Ulva: shown is the position of the island relative to western Scotland, and the location of the study site (dotted lines) on the southern coastline of Ulva (top left)

Fieldwork was carried out on the Isle of Ulva, located in the Inner Hebrides in Argyll and Bute, western Scotland (56° 28′ 39.18″ N, 6° 12′ 24.91″ W). Ulva is approximately 16km^2^ and is characterized by an altitudinal change in habitat types. At sea level, coastal herb-rich grasslands dominate with fens occurring intermittently throughout. Sub-montane basalt outcrops denote the upper limit of these grasslands, above which heather/bracken (*Calluna vulgaris*/*Pteridium aquilinum*) mosaics occur throughout the entirety of the higher altitudes of the island (e.g. 50–300 mamsl) of the island. On Ulva, transparent burnets are limited to sloping grasslands located on a small portion of the southern coastline shared by slender scotch burnets (*Z. loti*) and six-spot burnets (*Z. filipendulae*). A 1 km stretch of coastline occurring below tall basalt cliff faces was chosen as the study area (Fig. 1). This area was characterised by south, south-easterly and south-westerly facing grasslands (22–80 mamsl) that featured a steep incline (30–40º). All sites featured loose and unstable soil conditions, vegetated by a relatively herb-rich community with respect to the other habitats on the island.

### Moth trapping

A novel technique of pheromone-baited funnel traps was employed to collect data on spatio-temporal variation in the abundance of burnets. The application of pheromone-baited traps as an ecological monitoring tool has generally been limited to quantifying the presence of pest populations (Witzgall et al. 2010). Extending the utility of pheromone-baited traps to monitor rare and declining *Zygaena* populations, in addition to determining suitable habitat locations and habitat continuity, has been proposed previously (Burman et al. 2016; Larsson et al. 2003; Oleander et al. 2015); however, only one study on zygaenids has been published to date (Bergman et al. 2019). Here, we use pheromone traps in conjunction with traditional habitat surveying methods to examine the ecological drivers of burnet abundance.

Locations for placing funnel traps were derived from surveys and from anecdotal information regarding the local distribution of the species (pers. comms. J. Howard). Twenty-two Oecos Economy Funnel Traps baited with female transparent burnet pheromone (Priesner et al. 1984) were placed at 50 m intervals and numbered in sequence from east to west. Traps were baited on alternate days. On trapping days, traps were baited with the pheromone lure at 1800 h GMT, when most insect activity had ceased for that day. After 24 h, moths inside the trap and within a 5-m radius of the trap were counted and the captive specimens were released. On each trapping day, a Kestrel 1000 anemometer was used to record wind speed at each site for a duration of 10 s at a 0.4/s resolution. The pheromone lures were then removed from the traps until the following day to prevent excessive disruption to the breeding season.

All twenty-two traps were employed over a period of 15 days. Recording started prior to peak emergence of adult males. Throughout the entire field season, the study site was investigated every 3–4 days for the first flyers, and recording was initiated the day after the first observations of imago activity (28/06/2015). Data collection continued until 16/07/2015, at which point moths were neither observed in the field nor caught in the traps. Nine recording days were conducted in this timeframe, but the last 2 days were omitted from analysis due to low moth numbers (two and one moths were caught, respectively). Traps were located by use of a Garmin eTrex GPS receiver.

### Habitat assessment

As pheromone lures could attract individuals from a range of distances through targeted flight, catch-rates may represent the quality of local habitat in addition to the abundance and/or quality of habitat patches further afield. To account for this, variables were recorded at four different scales: 2-m radius, 25 m^2^ (3.53 m radius), 100 m^2^ (7.07 m radius), and at a 50-m radius (Table 1). For ease of data collection, intermediate scale habitat was assessed within square quadrats, whereas the variables at both the smallest and largest scales were best determined using circular radii. At the 2-m radius, the percentage cover of all plant species was recorded. At the 25 m^2^ scale, the following variables were recorded: number of blooming thyme forbs, mean vegetation height, percentage of bracken cover, the presence/absence of intersecting deer trails and the number of deer pellet groupings. Deer pellet groupings were defined as discrete concentrations of pellets aggregated within a 15 cm-radius. Vegetation height recorded at the 25 m^2^ was calculated by averaging 5 randomly placed drop-disc measurements within the 25 m^2^ plot, whereby a 30-cm radius cardboard disc, running free on measuring tape, was dropped vertically onto the vegetation (Ravenscroft and Young 1996). Bracken cover was calculated by summing the cover in each constituent square metre, which, themselves, were assessed with a 1 m^2^ quadrat. At the 100 m^2^ scale, the percentage cover of short-sward grassland, bracken and heath communities was recorded. Estimates of percentage cover were standardised by dividing the 100 m^2^ area into four 25 m^2^ quadrants. Visual estimates of cover were made for each quadrant, and pooled together to retrieve percentage cover at the entire 100 m^2^ scale. Observer consistency was maintained for all plots. Data collection at the 50-m radius scale was performed remotely via post-fieldwork GIS analysis in ArcGIS 10.1 (ESRI 2011). The proportions of short-sward grassland and bracken within the 50-m radius of each trap were recorded by digitizing ortho-rectified imagery (Online Resource 1). Additionally, the aspect (º from north), slope (º) and altitude (mamsl) of each trap location were derived from a digital elevation model of the study area.

**Table 1:** Independent variables recorded at trap locations respective to each scale: 2-m radius, 25 m^2^, 100 m^2^, and 50-m radius

Scale	Variables recorded
2-m radius	Percentage cover of all plant species (incl. bare soil)
	Shannon–Wiener plant diversity
25 m^2^	No. of blooming thyme forbs
	Percentage cover of bracken
	Mean vegetation height
	No. of deer pellet groupings
	Deer trail presence/absence
100 m^2^	Deer pellet presence/absence
	Deer trail presence/absence
	Percentage cover of bracken
	Percentage cover of heather
	Percentage of short-sward grassland
50-m radius	Aspect
	Slope
	Altitude
	Short-sward grassland cover (GIS supervised classification)

### Statistical analyses

Generalized linear mixed-effect models (GLMMs) were used to test the topographic, vegetative and deer-related effects on imago counts. Frequently, imagos were found clustered on the outer surface of the traps themselves. Since the number of imagos found on the outside surface of the traps and inside traps were strongly co-linear (correlation coefficient = 0.628), these counts were summed for each trap and used as the response variable, assuming a negative-binomial error distribution.

Variables recorded at each scale were analysed against total imago abundance separately, avoiding collinearity between similar variables at varying scales; the exceptions were altitude, aspect, slope and wind, which were included at every scale to account for topographic structure in the data. At the 2-m radius scale, twenty-one species/cover types (including bare soil and rock) were recorded; 11 of these species were sufficiently common to be used in analysis: velvet grass (*Holcus mollis*); tormentil (*Potentilla erecta*); bird’s foot trefoil (*Lotus corniculatus*); wild thyme; white clover (*Trifolium repens*); common heather; lady’s bedstraw (*Galium verum*); bell heather (*Erica cinerea*); fairy flax (*Linum catharticum*); *Fescue* spp. and *Agrostis* spp. *Fescue* spp. and *Agrostis* spp. were found to be closely associated and pooled together to form ‘short-sward’ grass cover, and common heather and bell heather were pooled into a ‘heath’ category as these two species were found exclusively as mosaics. Additionally, a Shannon–Wiener plant diversity index derived from these 11 species was included as a variable at the 2 m-radius scale. At the 25 m^2^ scale, vegetation height and percentage of bracken were found to be co-linear and, thus, only bracken cover as a percentage was used for analyses. Pellet groupings were analysed as categorical data: 0 pellet groups, 1 pellet groups, 2 pellet groups, and 3 or more pellet groups. Deer trails were included as a presence/absence category. To account for the site structure of the data and for changes in emergence patterns over the course of the flight season, site and recording day were included in the models as random effects.

To compare the explanatory value of models, model selection used Akaike’s information criterion (AIC) to rank models. Specifically, we considered for further inference all models with ΔAIC ≤ 6, unless they were more complex versions of nested models with lower AIC values (Richards et al. 2011). GLMMs were fitted with the ‘lme4’ package (Bates et al. 2015) in R 4.0.3 (R Core Team 2020 Team). Model fit was validated with the ‘DHARMa’ package (Florian 2020), and detailed in Online Resource 2. The proportion of explained variance by the fixed effects (marginal *R*^2^) and the fixed and random effects combined (conditional *R*^2^) was approximated for each model using the ‘r.squaredGLMM**’** function in the ‘MuMIN’ package (Barton 2020).

## Results

The highest number of males caught in a trap was 41. The highest total number of moths caught in all traps on a single day was 267 (Day 3). A total of four imagos were confirmed to have perished in traps throughout the recording period. Seven of the twenty-two traps featured deer trails within the 25 m^2^ scale. Deer pellet presence at the 25 m^2^ scale was recorded at seven traps, ranging from one to four pellet groupings. Tormentil was the most commonly found flower, found at > 20% cover at 6 sites, whereas white clover was the least prevalent, found at 5% cover or less at 18 sites. Velvet grass was the most prevalent species, found on all sites, occuring at > 20% cover at 17 sites.

**Fig. 2:**
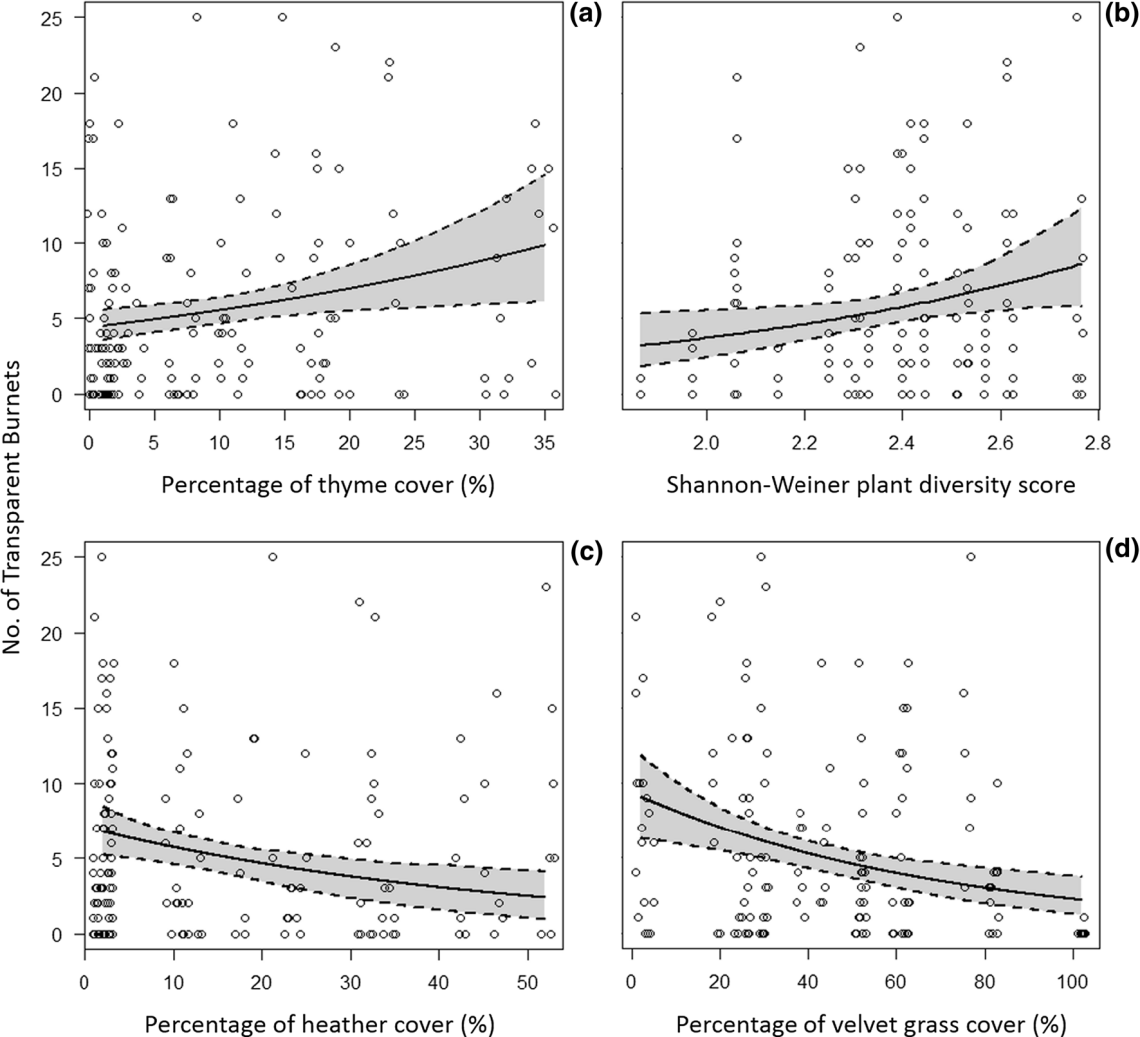
The main effects of **a** thyme cover, **b** Shannon–Wiener plant diversity, **c** heather cover, and **d** velvet grass cover on the abundance of *Zygaena purpuralis* imagos at the 2-m radius scale. Solid line represents the mean main prediction of the variable of interest whilst restricting all other predictors retained in the best model at mean values. Shaded areas delineated by dotted lines represent CIs calculated from bootstrapping the respectice main prediction (*n* = 1000). Data points are jittered to show overlapping values

Of the 13 different variables tested at the 2-m radius scale, 4 were retained in the best model: vegetation diversity, and the cover of heather, velvet grass and thyme (Table 2). The total number of imagos found in traps was positively related to thyme cover and vegetation diversity, and was negatively influenced by heather and velvet grass cover (Fig. 2, Table 2).

**Table 2:** GLMM outputs of transparent burnet imago abundance tested against variables at 2-m radius, 25 m^2^ and 100 m^2^ scales

Scale		*AIC*	*ΔAIC*	*ndf*	*cdf*	*mr*^*2*^	*cr*^*2*^	*LL*
2-m radius	SHANNON + HEATH + VGRASS + THYME	804.9	0	8	139	0.203	0.632	− 393.924
25 m^2^								
M1	PELLETS + TRAIL + BRACKEN +	635.1	0	9	133	0.323	0.573	− 307.879
M2	PELLETS + TRAIL + BRACKEN + WIND	637.4	2.31	10	132	0.323	0.573	− 307.877
100 m^2^	BRACKEN	525.6	0	5	142	0.056	0.627	− 257.59

Displayed models include all the retained models with ∆AIC < 6 for each scale, whilst omitting models that represented more complex versions of retained nested models. The null model was retained for the 50 m-radius model. All models include random intercepts for recording day and site. M1 and M2 indicate model 1 and model 2, respectively

*SHANNON represents Shannon–Wiener plant diversity. HEATH, VGRASS, THYME and BRACKEN represent heath, velvet grass, thyme and bracken % cover, respectively. PELLETS represents pellet presence as a count variable, and TRAIL represents deer trail presence as a dichotomous categorical variable. WIND represents wind speed. Numerator and denominator degrees of freedom are represented by ‘ndf’ and ‘ddf’, respectively. Marginal and conditional r2 values are represented by ‘mr2’ and ‘cr2’, respectively. Log-likelihood is denoted by ‘LL’

**Fig. 3:**
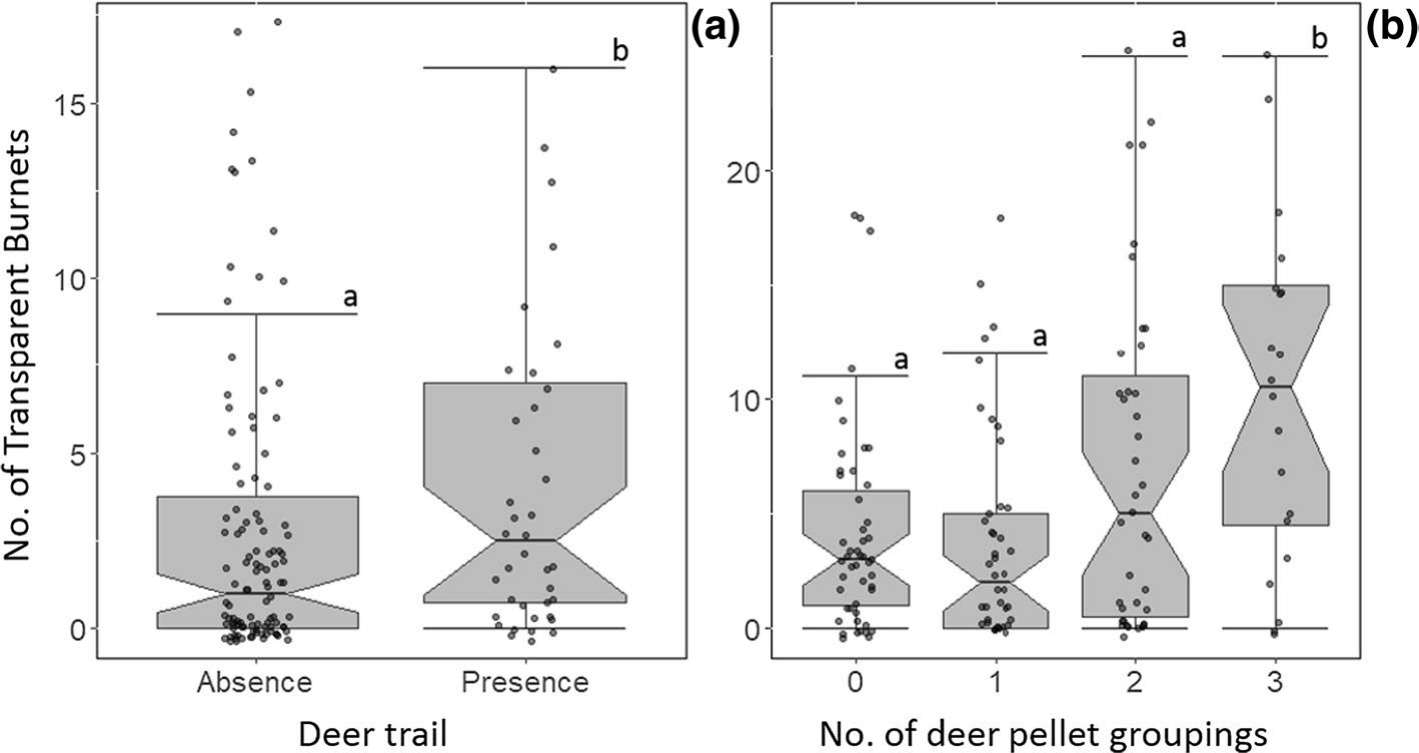
The total abundance of transparent burnet imagos found at trap sites in relation to **a** deer trail presence and **b** the number of pellet groups within the 25 m^2^ scale. Boxes represent the interquartile range surrounding the median (horizontal line inside boxes), notches indicate confidence interval respective to the median. Whiskers indiciate the 75th and 25th percentile, respectively. Data points are jittered to show overlapping values

**Fig. 4:**
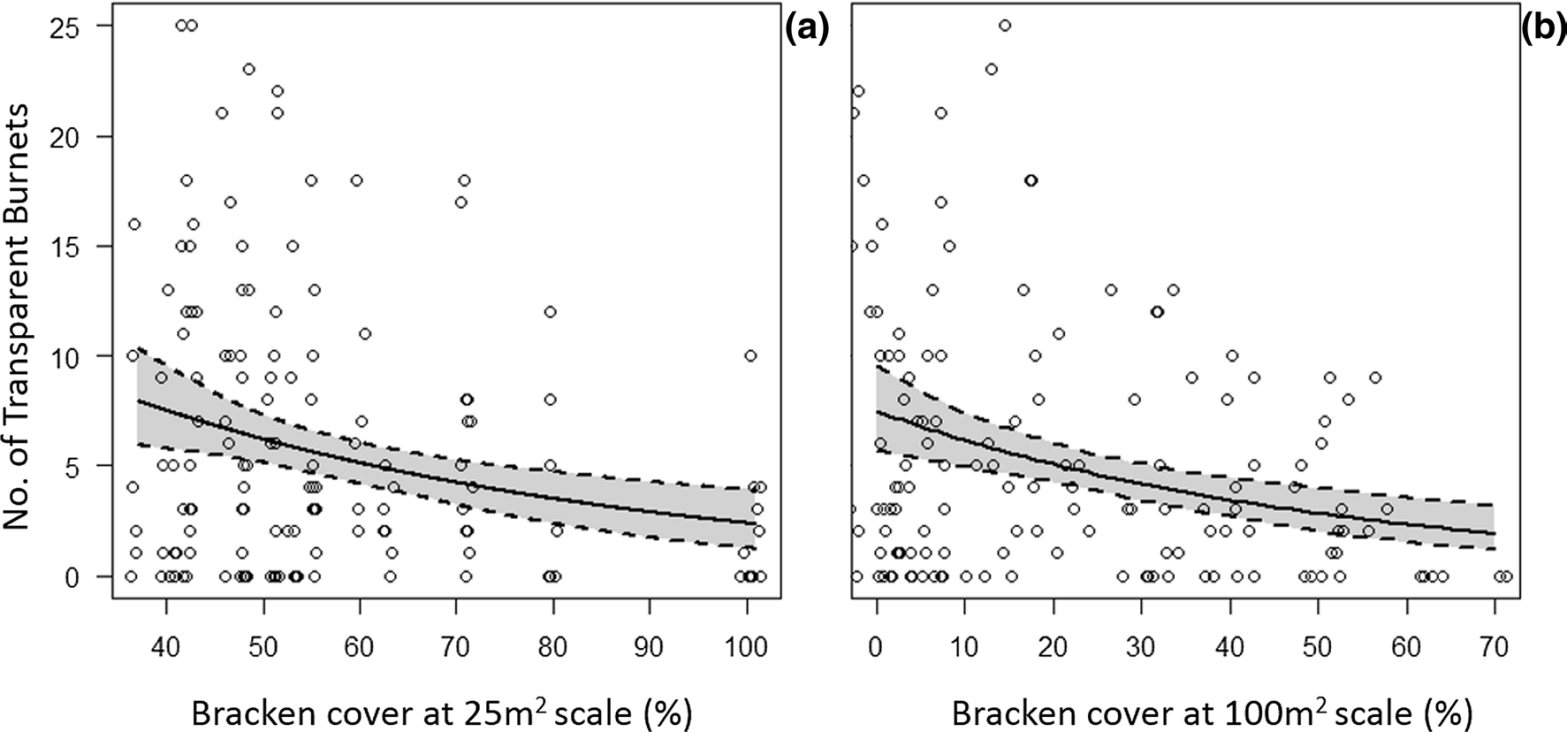
The main effects of bracken cover at the **a** 25 m^2^ scale and at the **b** 100 m^2^ scale on the abundance of transparent burnet imagos. Solid line represents the mean main prediction of the variable of interest whilst restricting all other predictors retained at mean values. Dotted lines represent CIs calculated from bootstrapping the respective main prediction (*n* = 1000). Data points are jittered to show overlapping values

At the 25 m^2^ scale, deer pellet abundance, deer trail presence and bracken cover were retained in the best model (Table 2); however, an alternative model that retained average wind-speed instead of bracken cover was also selected (∆AIC = 2.31, Table 2), suggesting that bracken cover is less influential in predicting imago abundance than pellet abundance or deer trail presence. Imago abundance was positively influenced by deer pellet abundance and by the presence of deer trails (Fig. 3; Table 3). Both average windspeed and bracken cover shared a negative relationship with imago abundance (Table 3). Bracken cover was also retained in the best model at the 100 m^2^ scale (Table 2) and shared a negative relationship with imago abundance (Fig. 4; Table 3). No other variables were retained at the 100 m^2^ scale, and no variables were retained at the 50-m radius scale (null ∆AIC = 0).

**Table 3:** Coefficient estimates, standard errors, and *p*-values for the variables retained in the selected models for predicting transparent burnet imago abundances at the 2-m radius, 25 m^2^, and 100 m^2^ scales

Scale		Coefficient estimate	Standard error	*p* value
2-m radius	*Intercept*	2.191	0.371	
	*SHANNON*	0.792	0.392	0.045*
	*THYME*	0.033	0.009	> 0.001***
	*HEATH*	− 0.024	0.006	> 0.001***
	*VGRASS*	− 0.015	0.004	> 0.001***
25 m^2^ M1	*Intercept*	1.999	0.427	
	*PELLET3*	0.62	0.269	0.023*
	*TRAIL*	0.5	0.187	0.009**
	*BRACKEN*	− 0.014	0.005	0.008**
25 m^2^ M2	*Intercept*	2.111	0.544	
	*PELLET3*	0.602	0.277	0.031*
	*TRAIL*	0.517	0.188	0.007**
	*BRACKEN*	− 0.015	0.006	0.011*
	*WIND*	− 0.052	0.117	0.66
100 m^2^	*Intercept*	0.765	0.376	
	*BRACKEN*	− 0.018	0.005	0.008**

M1 and M2 indicate model 1 and model 2, respectively. Statistically significant results are highlighted in bold font for *p*-values with asterix codes indicating: > 0.001***, > 0.01**, > 0.05*

## Discussion

We found that relatively few local-scale ecological factors influence the local abundance and habitat use of transparent burnets. Positive drivers of burnet abundance included, at a very local scale, high vegetation diversity and the abundance of the preferred adult food plant, thyme. By contrast, velvet grass and heather on a local scale and high bracken cover in the wider area all had negative impacts on burnet abundance. Importantly, we also found that red deer trails and habitat use could be an important determinant of habitat suitability for burnets. We discuss these findings with respect to investigating the importance of trophic interactions and non-trophic interactions (via ecosystem engineering) on transparent burnet abundance.

### Trophic interactions

Transparent burnets feed predominantly on the larval host plant, thyme, but small proportions of alternative nectar sources also contribute, such as bird’s foot trefoil, tormentil, and white clover (Bourn 1995; Öckinger 2008). This explains the association of high trap counts with thyme percentage cover and overall vegetation diversity, given that the other forbs listed are the major contributors to diversity in grasslands (Howe 1994), but also explains the lack of relationship found with the cover of any specific forb species. Similarly, the habitat preferences of crepuscular burnet (*Z. carniolica*) are determined by relatively few nectar plants (Binzenhöfer et al. 2005) and according to Thomas et al. (1992), habitat suitability for most insect species are driven by factors important to the larvae stages as opposed to the adult. Indeed, larval host plant availability has been shown to be a more important driver of burnet imago abundance than habitat connectivity or abundance of nectar plants (Bergman et al. 2019). For example, the abundance of six-spot burnet (*Z. filipendulae*) and narrow-bordered five-spot burnet (*Z. lonicerae*) imagos have been found to be largely dependent on larval resource availability (Öckinger 2008). Additionally, the dispersion of the new forest burnet (*Z. viciae*) was found to be unrelated to the distribution of adult nectar sources (Franzén and Nilsson 2012). This rationale may explain the lack of explanatory power of variables found at the larger scales; habitat suitability at the 7.07-m (100 m^2^) and 50-m radii was based on the cover of herb-rich habitat instead of assessments of the larval host plant thyme.

Velvet grass is a tall, obstructive gramminoid that provides little value to transparent burnets. Furthermore, transparent burnets are thought to favour low-lying, early-successional grasslands, and velvet grass is generally associated with grasslands of a later seral stage (Ovington 1953). Whilst a source of nectar, heather species also represent a divergence from suitable habitat and reduce the availability of low-laying forbs and grasses in sheltered hollows in the ground. Nevertheless, heather is commonly found with suitable herb-rich grasslands and the negative relationship found indicates that, like the crepuscular burnet (*Z. carniolica*), transparent burnets represent a highly stenotypic taxon (Habel et al. 2012). These trophic effects were only relevant at a very local scale. Transparent burnets typically exhibit limited mobility (Bourn 1995; Clausen et al. 2001) and, upon locating an ideal forb-rich patch with ample thyme, are likely to remain locally take advantage of these resources.

Bracken proved to be a strong deterrent of transparent burnets at broader scales. This large fern is typical of sub-climax communities (Marrs and Hicks 1986), representing habitat conditions that are inconsistent with the needs of transparent burnets. Bracken might also present obstructive architecture in the environment, limiting the number of moths that can access a given trap. During the time that fieldwork took place, bracken was at peak growth, and can reach up to 1.5 m in height (Marrs et al. 2000). As weak flyers, transparent burnets may struggle to navigate through bracken thicket. By limiting transparent burnet dispersal, bracken cover may fragment suitable habitat into isolated patches that experience limited migration, thus enforcing a metapopulation structure (Hill et al. 1996). As burnets cannot colonise new areas prior to the establishment of their foodplant and microclimatic conditions (Zagrobelny et al. 2019), populations may be highly susceptible to the adverse effects of habitat fragmentation (Habel et al. 2012).

Transparent burnet abundance was positively associated with the highest counts of deer pellets. Pellet abundance is a proxy for grazing pressure (Limpens et al. 2020), which suggests that a relatively high pressure of deer grazing is beneficial in maintaining an early-successional stage suitable for transparent burnet larval development. These findings contribute to the increasingly acknowledged conservation benefits of red deer herbivory (Virtanen et al. 2002; Mysterud 2006; Smolko et al. 2018). Various butterfly species such as small copper (*Lycaena phlaeas*) and grayling (*Hipparchia semele*) have also been shown to benefit from extensive levels of red deer grazing (WallisDeVries and Raemakers 2001) and the implementation of red deer grazing as a viable method for conserving semi-natural grasslands has been advocated by Riesch et al. (2019). The slender scotch burnet moth (*Z. loti*), which co-occurs with transparent burnets in many locales in Scotland and shares similar habitat requirements, also benefits from high degrees of livestock grazing pressure (Ravenscroft and Young 1996). As livestock grazing is absent from our study site, the red deer grazing regimes present appear to be an effective surrogate for sustaining burnet habitat via intensive grazing. The herb-rich *Agrostis-Festuca* grasslands occupied by transparent burnets and slender scotch burnets are grazed to a similarly high effect by both red deer and hill sheep alike, as these grasslands represent forage of relatively high levels of digestibility and nutrient content compared to the alternative upland habitats present (Charles et al. 1977).

### Non-trophic interactions

In addition to the trophic effects described above, our study also identified a clear non-trophic interaction, in that red deer may be playing an active role in provisioning specific habitat requirements for transparent burnets. Deer trails represent a specific type of deer activity; the formation of trails specifies the spatial nature of concentrated and repeated locomotive behaviour (O'Neill 2017). It is not surprising that transparent moths were found to be associated with deer trails, as these features provide a key habitat requirement: bare soil. Transparent burnets, along with related species, benefit from bare soil as the darker surface absorbs more heat than green vegetation, and complements the conditions of a warm microclimate favoured by various zygaenids (Ravenscroft and Young 1996; Streitberger and Fartmann 2015). Additionally, female transparent burnets exhibit strong selectivity when laying egg batches and prefer sites close to exposed soil (Bourn 1995). The formation of deer trails is caused by daily altitudinal descents/ascents and, therefore, trails are found more frequently on sloping terrain (Clutton-Brock et al. 1982). The slopes inhabited by transparent burnets are used by deer for this purpose; in doing so, the deer maintain an aspect of the microhabitat conditions necessary for the persistence of transparent burnets.

Transparent burnets have also been recorded to benefit from trampling of mosses and grasses by livestock, as compressed vegetation retains more heat (Bourn 1995). Additionally, low-lying herbs such as thyme benefit from patches of exposed soil caused by animal trampling as disturbance favours shade-avoiding herbs by oppressing tall-growing competition (Fleischer et al. 2013). The diurnal migration of deer that pass through these south-facing slopes may be similarly trampling over the vegetation and further maintaining suitable habitat for transparent burnets. Similarly, transparent burnets have been shown to benefit from soil-disturbing ecosystem engineering in Central Europe; anthills in semi-natural grasslands function as important microhabitats for transparent burnets as these structures feature bare soil and a high cover of thyme and other low-lying forbs (Streitberger and Fartmann 2015).

### Pheromones and monitoring techniques

Prior to this study, pheromone-baited live traps have not been used to monitor *Zygaena*, and this research also represents the first use of pheromones synthesised specifically for transparent burnets (Bergman et al. 2019). Pheromone-baited live traps are an attractive tool as current sampling methods such as sweep-netting or pitfall traps can be time-consuming and are dependent on taxonomic expertise (Oleander et al. 2015). However, it is important to recognise assumptions inherent in this novel method, and recognise any potential negative effects of exposing species of conservation concern to pheromones.

During the flight period, male burnets rely on olfactory and visual cues to detect and locate the most proximate females (Hofmann and Kia-Hofmann 2010). Once female activity is located, males remain in the close vicinity for the remainder of the flight season (Hofmann and Kia-Hofmann 2010; Koshio and Hidaka 1995). Pheromone-baited traps do not adversely affect mate location (Oleander et al. 2015), and males have been shown to avoid targeted flight towards traps altogether and instead form concentrations around more proximate females (Bergman et al. 2019; Ryrholm, unpublished). Consequently, the ability of pheromone-baited traps to attract male imagos may decline at larger spatial scales, whereby pheromone signals from areas of female activity are more likely to be encountered first. The lack of explanatory power of variables measured at the larger scales in the current study could be attributed to these limitations.

When pheromones are used as a tool to investigate the abundances or densities of a species, there is a potential bias that may arise from solely sampling male imagos. Gendered differences in the residence times and life expectancies of Lepidoptera have been documented previously, for example, Gall (1984) found that females lived longer and emigrated from the natal site at a much older age than males. Interestingly, this suggests that sampling from one gender of the population may be further confounded by age. For instance, male heath fritillary (*Mellicta athalia*) have been shown to exhibit a reduction in range size from 120 to 60 m as they aged, whereas females movement increased from 30 to 100 m with age (Warren 1987). For transparent burnets, the life histories of both genders are similar (Bourn 1995), and sampling males is likely a reliable estimate of overall population abundance. However, with respect to the general utility of pheromone traps as an ecological monitoring tool, an understanding of potential sex-based differences in a species of interest should be an important prerequisite in making population-level assumptions.

## Conclusions

Our findings have provided evidence that the abundance of transparent burnet moths is simultaneously affected by direct and indirect trophic interactions with red deer, both of which should be addressed when regarding the conservation of the species. The encroachment of bracken upon the slopes seems to be creating a mosaic of grassland patches, threatening the persistence of the transparent burnet population through patch isolation and habitat fragmentation. As weak flyers, transparent burnets may be particularly susceptible to the problems of persisting in an increasingly disjoint metapopulation structure (Franzén and Ranius 2004; Dieker et al. 2013). Therefore, management should focus not only on removal of bracken, but should also consider the continuity of grassland habitat and avoid the formation of isolated patches by encroaching bracken. Since the patterns of free-roaming red deer activity are currently maintaining the precise habitat conditions required for transparent burnets, conservation efforts should focus on ensuring that the patterns of tourist activity on the island do not induce behavioural changes and habitat usage in the red deer population to the detriment of transparent burnet habitat. Understanding how direct and indirect trophic determinants affect a species allows conservationists to prioritise efforts and use the behaviour of ecosystem engineers to achieve desired outcomes.

## Supplementary Information

Below is the link to the electronic supplementary material.

Supplementary file1 (PDF 107 KB)

Supplementary file2 (PDF 365 KB)

## Data Availability

The datasets generated during and/or analysed during the current study are available from the corresponding author on reasonable request.

## Code availability

The code generated during and/or analysed during the current study are available from the corresponding author on reasonable request.
