# Supplementary material for: The importance of direct and indirect trophic interactions in determining the presence of a locally rare day-flying moth
Source: Oecologia. 2022 Jan 8;198(2):531–42. doi: 10.1007/s00442-021-05100-9 (PMC8858923; doi:10.1007/s00442-021-05100-9)
Supplement: Supplementary file 1 — Supplementary file1 (PDF 107 KB) [file 442_2021_5100_MOESM1_ESM.pdf]

Online Resource 1. Post-fieldwork ArcGIS analyses

Data collection at the 50-m radius scale was performed remotely via post-fieldwork GIS analysis; the amount of short-sward cover within a 50-m radius of each trap was calculated in two steps. First, the 'Buffer' tool was used to create buffer polygons around each trap, specified in extent to 50-m radius. Second, short-sward habitat was digitized by a supervised classification process. Specifically, a multi-band RGB high-resolution TIFF file (4800x3156) of Ulva was sourced from Google Earth (Google Inc, 2009). This multi-band image was exported into ArcGIS 10.1. (ESRI, 2011), and georeferenced onto the British National Grid system. Image classification tools (enabled with the Spatial Analyst extension) allowed for the creation of signature files, which are examples of the spectral signatures (specific RGB combinations) of the short sward habitat in the image file, collected by the user. The final short sward cover raster was produced from extrapolating these example signature files across the entire image i.e. raster cells were created from assigning the correct signature files depending on the spectral signature of the underlying image file in a given area. Raster cell size was 5m. Finally, the 'Spatial statistics' tool was used to calculate the number of short-sward raster cells pertaining to the habitat classification raster that overlapped with each 50-m buffer polygon. These values were then attributed to each respective trap. The aspect, slope and altitude (5m resolution) of the terrain at each trap location were retrieved using the 'Extract to Point' tool. Using the 'Surface' toolset, rasters for aspect and slope were were derived from a digital elevation model of the site. Alongside altitude (mamsl) data present within the digital elevation model, aspect (° from north) and slope (°) were attributed to each respective trap location to provide variables for altitude, slope, and aspect.
