# Supplementary material for: The importance of direct and indirect trophic interactions in determining the presence of a locally rare day-flying moth
Source: Oecologia. 2022 Jan 8;198(2):531–42. doi: 10.1007/s00442-021-05100-9 (PMC8858923; doi:10.1007/s00442-021-05100-9)
Supplement: Supplementary file 2 — Supplementary file2 (PDF 365 KB) [file 442_2021_5100_MOESM2_ESM.pdf]

Online Resource 2. Reporting variable collinearity and testing for over/under-dispersion for models

COLLINEARITY OF VARIABLES

In modelling the potential effects of multiple variables (particularly at the 2m-radius scale given that numerous plant cover percentages were tested for simultaneously) removing potentially collinear variables was a critical step in the statistical analyses. The steps for detecting and dealing with collinearity outlined by Zuur et al. (2010) were followed for this study, and the results are presented on page 1; Table S1 and Table S2 details collinearity for the 2m-radius and 25m^2 variables, respectively. Collinearity of variables is not reported for the 100m^2 model as only one variable was retained at this scale.

Table S1. Collinearity between variables at the 2m-radius scale was tested for via variance inflation factor (VIF) values, whereby a value of 3 was determined to be the threshold (Zuur et al., 2010). Fescue *spp*. and Agrostis *spp*. exhibited collinearity (VIF >3), and these were pooled into a 'short-sward grass' variable as these two species described the grassland community of the study site.

**Variable**	**VIF**
SHANNON	1.634
HOLCUS	2.198
LOTUS	1.547
THYME	1.527
CATHAR	1.336
REPENS	1.468
GALIUM	1.075
HEATH	2.297

Table S2. Collinearity between variables at the 25m^2 scale was tested for via variance inflation factor (VIF) values, whereby a value of 3 was determined to be the threshold (Zuur et al., 2010). Vegetation height and bracken were found to be collinear (VIF >3), and height was removed.

**Variable**	**VIF**
BRACKEN	1.782
PELLET	2.2
TRAIL	1.423
WIND	1.21

MODEL VERIFICATION FOR HETEROSCEDASTICITY

Heteroscedasticity in each of the final models was tested for using a combination of visual diagnostics and test functions found in the 'DHARMa' package (Florian, 2020), which is specifically designed for producing residual diagnostics for fitted generalised linear mixed models. Diagnostics for the fitted models reported in this study are reported throughout pages 2-4.

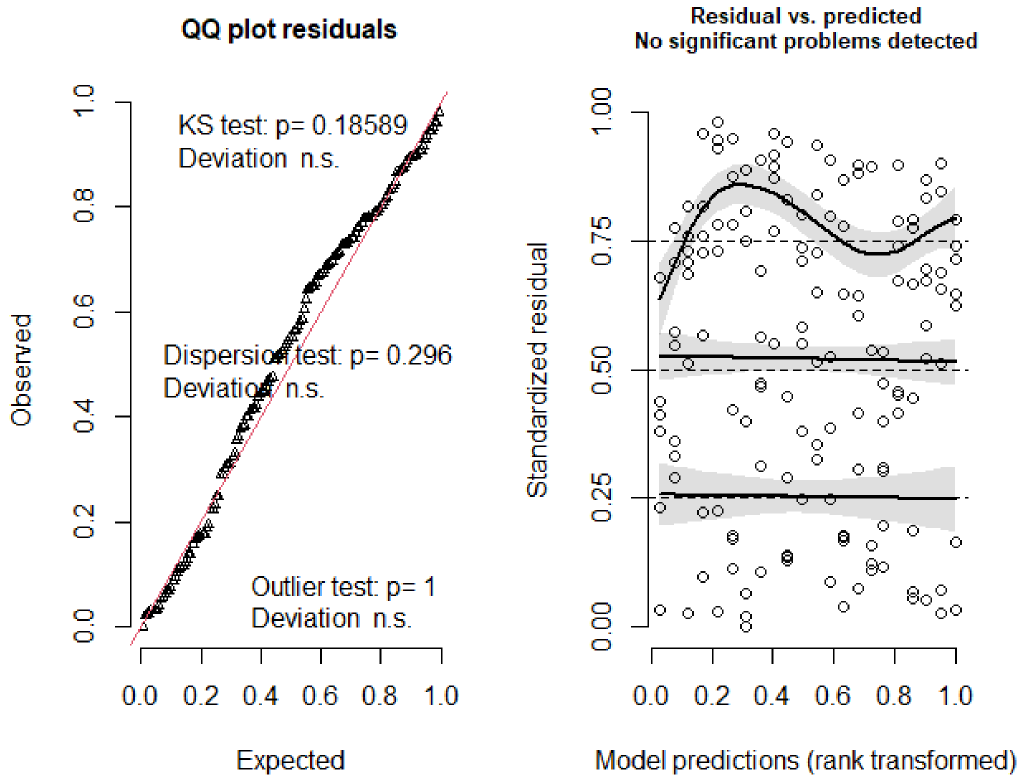

Figure S1. Residual diagnostics for the 2m-radius model generated from the 'DHARMa' package (Florian, 2020) in R Statistical Software (version 4.0.3, R Development Core Team 2012): a quantile-quantile probability plot of model residuals plotted against the expected distribution, overlaid with p-value results from Kolmogorov-Smirnov (KS), dispersion, and outlier tests; and model residuals plotted against model predicted values (right).

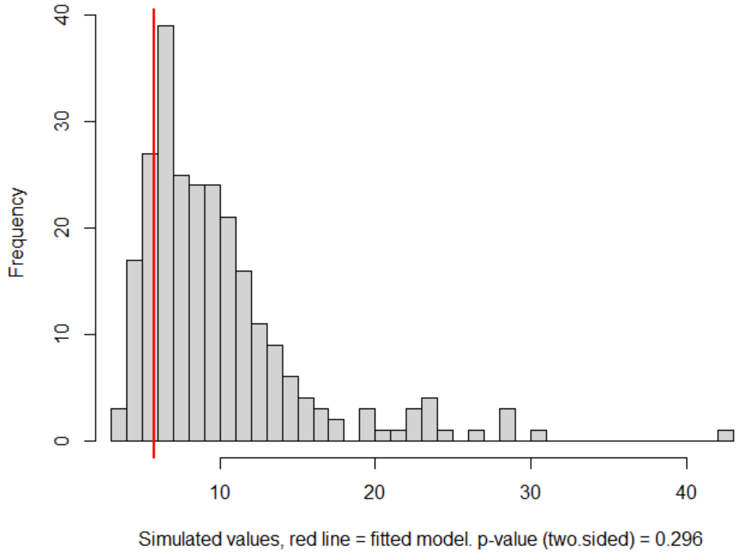

Figure S2. Targeted non-parametric over-dispersion test diagnostic for the 2m-radius model generated from the 'DHARMa' package (Florian, 2020) in R Statistical Software (version 4.0.3, R Development Core Team 2012). Observed residuals are plotted against the simulated residuals, overlaid with a p-value.

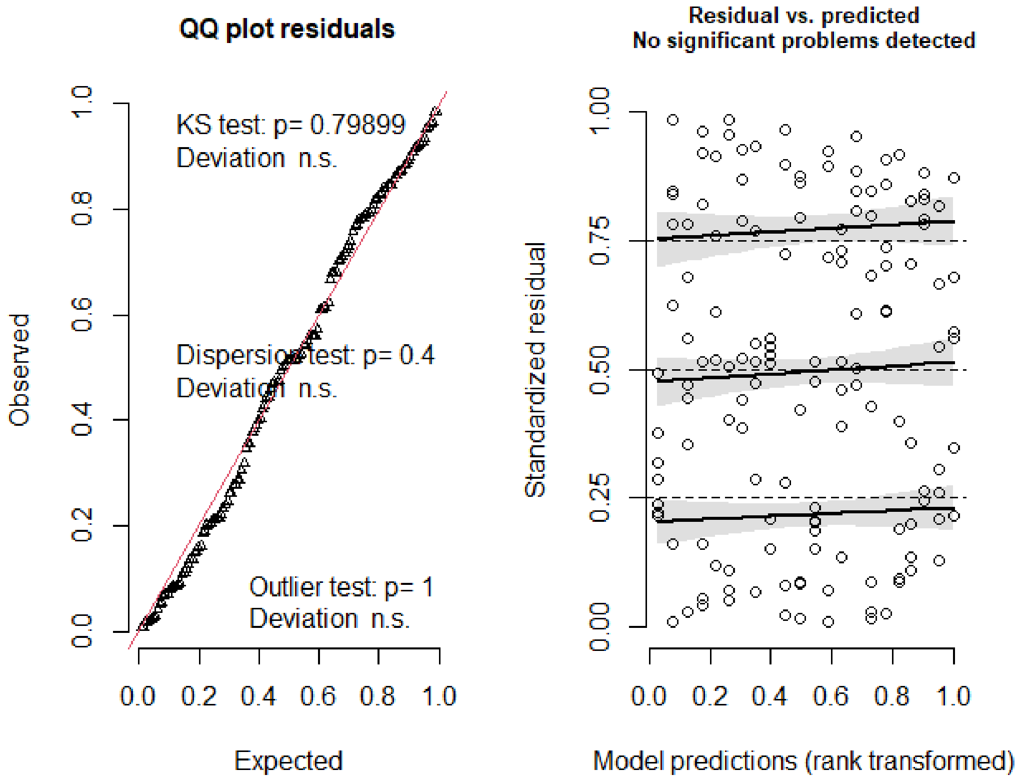

Figure S3. Residual diagnostics for the 25m^2 model generated from the 'DHARMa' package (Florian, 2020) in R Statistical Software (version 4.0.3, R Development Core Team 2012): a quantile-quantile probability plot of model residuals plotted against the expected distribution, overlaid with p-value results from Kolmogorov-Smirnov (KS), dispersion, and outlier tests; and model residuals plotted against model predicted values (right).

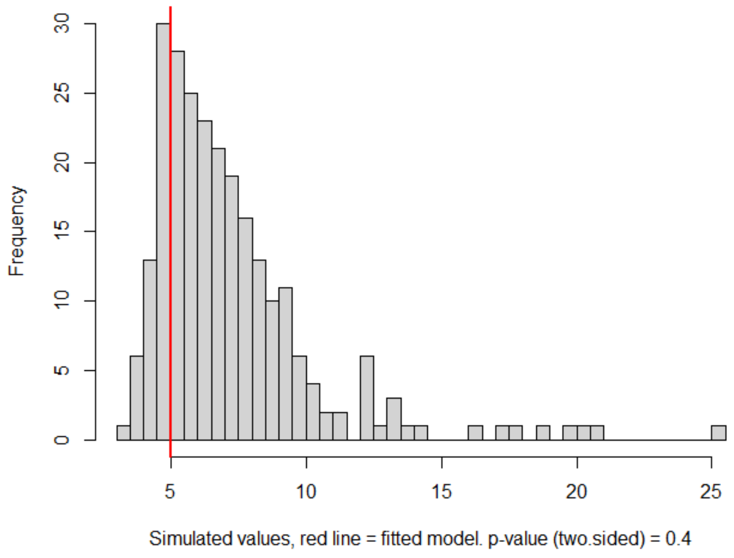

Figure S4. Targeted non-parametric over-dispersion test diagnostic for the 25m^2 model generated from the 'DHARMa' package (Florian, 2020) in R Statistical Software (version 4.0.3, R Development Core Team 2012). Observed residuals are plotted against the simulated residuals, overlaid with a p-value.

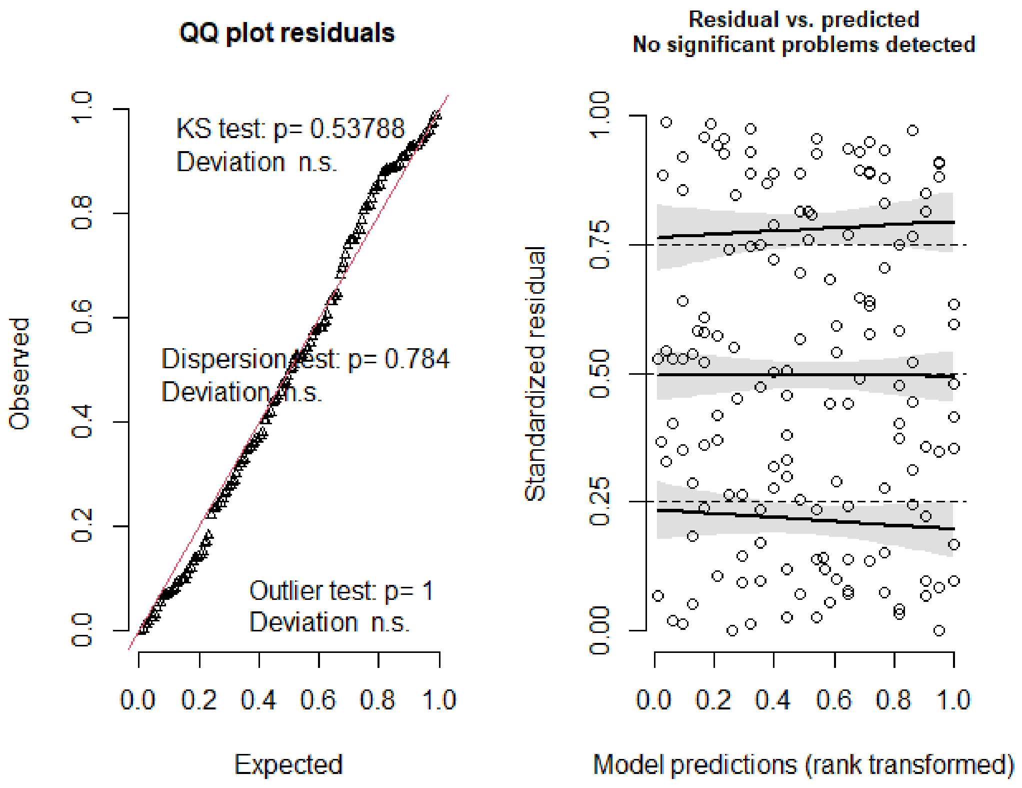

Figure S5. Residual diagnostics for the 100m^2 model generated from the 'DHARMa' package (Florian, 2020) in R Statistical Software (version 4.0.3, R Development Core Team 2012): a quantile-quantile probability plot of model residuals plotted against the expected distribution, overlaid with p-value results from Kolmogorov-Smirnov (KS), dispersion, and outlier tests; and model residuals plotted against model predicted values (right).

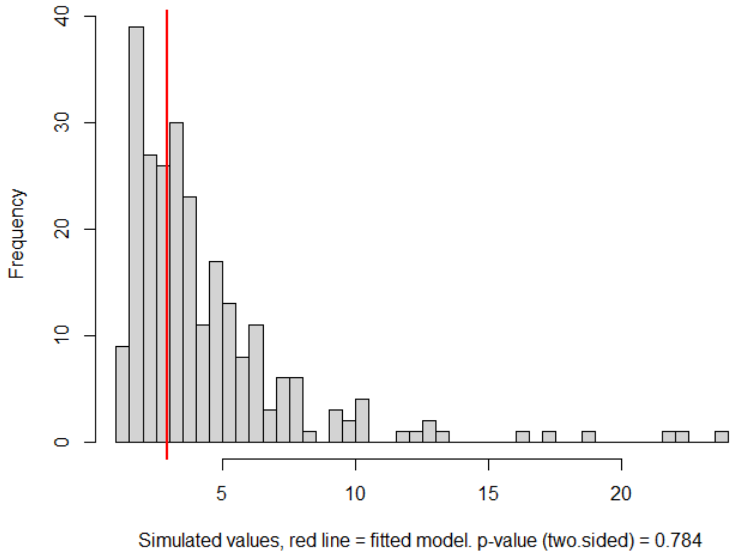

Figure S5. Targeted non-parametric over-dispersion test diagnostic for the 100m^2 model generated from the 'DHARMa' package (Florian, 2020) in R Statistical Software (version 4.0.3, R Development Core Team 2012). Observed residuals are plotted against the simulated residuals, overlaid with a p-value.

APPENDIX S1 REFERENCES

FLORIAN, H. 2020. DHARMa: Residual Diagnostics for Hierarchical (Multi-Level /Mixed) Regression Models. R package version 0.3.3.0. https://CRAN.R-project.org/package=DHARMa

OLEKSY, R.Z., AYADY, C.L., TATAYAH, V., JONES, C., HOWEY, P.W., FROIDEVAUX, J.S., RACEY, P.A. AND JONES, G., 2019. The movement ecology of the Mauritian flying fox (Pteropus niger): a long-term study using solar-powered GSM/GPS tags. *Movement ecology*, *7*(1), pp.1-12. https://doi.org/10.1186/s40462-019-0156-6

ZUUR, A.F., IENO, E.N. AND ELPHICK, C.S. (2010), A protocol for data exploration to avoid common statistical problems. Methods in Ecology and Evolution, 1: 3-14. https://doi:10.1111/j.2041-210X.2009.00001.x
